# Impact of squatting on selected cardiovascular parameters among college students

**DOI:** 10.1038/s41598-024-56186-z

**Published:** 2024-03-07

**Authors:** Uchechukwu M. Chukwuemeka, Chimdindu P. Benjamin, Chigozie I. Uchenwoke, Uchenna P. Okonkwo, Anthony C. Anakor, Stephen S. Ede, Ayodeji A. Fabunmi, Ifeoma A. Amaechi, Uzoamaka N. Akobundu

**Affiliations:** 1 Department of Medical Rehabilitation (Physiotherapy), Nnamdi Azikiwe University, Awka, Anambra State Nigeria; 2 Department of Medical Rehabilitation, University of Nigeria, Enugu, Enugu State Nigeria; 3 Department of Physiology, Nnamdi Azikiwe University, Awka, Anambra State Nigeria; 4 School of Sports and Health Sciences, University of Central Lancashire, Preston, UK; 5 Department of Physiotherapy, College of Medicine, University of Ibadan, Ibadan, Nigeria

**Keywords:** Physiology, Medical research

## Abstract

Squatting is an active posture test used in assessing baroreflex sensitivity, and the array of patients a physiotherapist handles may benefit from this test to avoid the adverse effects of exercise. Therefore, this study is designed to evaluate the effect of squatting on heart rate and blood pressure among undergraduate students. 35 males (mean age = 22.94 ± 1.846) and 40 females (mean age = 22.28 ± 2.075) participated in this experimental study. Demographic data and baseline cardiovascular parameters (blood pressure and heart rate) were taken before exercise. The exercise protocol, the squatting stress test, was done for 2 min, after which post-exercise blood pressure and heart rate were taken at one minute each. A repeated measure ANOVA and independent t-test were used to analyse the difference at the 0.05 alpha level. It was found that there was a significant difference between pre-exercise in lying and squatting post-exercise blood pressure and heart rate in the first and second minutes (*p* < 0.01), pre-exercise in lying and standing post-exercise blood pressure and heart rate in the first and second minutes (*p* < 0.01), pre-exercise in standing and standing post-exercise blood pressure and heart rate in the first and second minutes (*p* < 0.01), and pre-exercise in standing and squatting post-exercise blood pressure and heart rate in the first and second minutes (*p* < 0.01). Also, there was a significant difference in pre-exercise heart rate between lying and standing (*p* < 0.05) and not between the first minute and second minute post-squatting or standing exercise systolic blood pressure (*p* = 0.588) or diastolic blood pressure (*p* = 0.22–1). In conclusion, squatting trials among undergraduates revealed some statistically significant changes, especially between the cardiopulmonary parameters obtained in a standing position compared to lying and those measured after one minute. Therefore, caution should be observed when administering exercises that require changes in posture.

## Introduction

Squatting is an active posture test for baroreflex sensitivity^1^. It is also a core exercise for various purposes, including strengthening the leg and back muscles and core strengthening^2^. The squat is one of the most commonly used resistance exercises for performance and health because of its biomechanical and neuromuscular similarities to a wide range of athletic and everyday activities^3–7^. Squatting yields and generates a quick increase in cardiac output and arterial blood pressure which is attributed to an increase in venous return from compression of leg veins accompanied by an immediate decrease in heart rate and forearm vascular resistance which are due to activation of cardiopulmonary and arterial baroreflexes^8^. The shift from squatting to standing thus causes an effect of major orthostatic stress, leading to rapid and large changes in arterial blood pressure (BP) and heart rate, allowing an exact and accurate baroreflex assessment^1^. A squat programme is often measured by the external and internal load, which is the number of repetitions performed. These are the specific variables to quantify the external load, while different physiological and perceptual variables like the heart rate (HR), blood pressure (BP), and subjective scales of the rate of perceived exertion (RPE) quantify the internal load^9^.

If squatting is not carried out well, it can lead to injury and cause the non-targeted muscle to be activated and trained; the poor exercise technique will limit the actualization of performance gains and training goals^10^. Squat performance can be said to be influenced by certain factors, which include an individual's physical characteristics, such as leg length ratio and joint flexibility^11,12^. The loading of tissue to prevent and avoid injury and for adaptation is vital^2^.

Cardiovascular parameters are measures utilised in the assessment of cardiac responses, as seen in heart rate and blood pressure^13^. Studies have shown that as exercise is initiated and its intensity increases, there is a marked increase in oxygen demand by the body, especially the working muscles, giving rise to increased cardiac output by increasing stroke volume and heart rate as well as an increase in peripheral oxygen difference^14^. Blood pressure depends on cardiac output and peripheral vascular resistance; systolic blood pressure rises with increasing cardiac output, while diastolic blood pressure remains the same or is moderately decreased^14^. Researchers have shown that a decrease in diastolic blood pressure after exercise may be caused by a decrease in peripheral resistance by producing vasodilatation through an accumulation of metabolites like carbon dioxide and hydrogen ions^15^. It has also been reported that the cardiovascular response to squatting largely depends on factors such as volume of muscle mass, exercise duration, intensity, number of repetitions, and total workload^16^.

Few studies done in the past used squatting positions to evaluate patients and also as a therapeutic means to counteract the fall in blood pressure in patients suffering from dizziness and orthostatic hypotension or presenting pre-syncope symptoms, such as soon after exercise^1,17^. Tschakovsky et al.^18^ concluded that during rising from squatting, stroke volume, cardiac output, and mean arterial pressure all increased significantly, and heart rate decreased significantly. A study also used the squatting position as a diagnostic tool to evaluate blood pressure regulation^2^. However, there is a dearth of work in the literature that has evaluated the amount of change in cardiovascular parameters that would occur per change in position with time among undergraduate students within the West African context. Therefore, this study is designed to evaluate the effect of squatting on heart rate and blood pressure among undergraduate students.

## Methods

### Participants

Experimental studies of 75 apparently healthy undergraduate students (35 males and 40 females) with a mean age of 22.61 ± 1.96 were conducted to examine the study objective. The population for this study comprised undergraduate students of the Faculty of Health Science, Nnamdi Azikiwe University, Nnewi Campus. They were recruited through consecutive sampling techniques. The effect size of 0.15 was ascertained using G*Power at a sample size of 75, power of 0.95, and alpha of 0.05. Students with any disability limiting their ability to exercise and those who have a history of cardiovascular and respiratory diseases were excluded from the study.

Ethical approval for this study was granted by the Ethical Committee of Nnamdi Azikiwe University Teaching Hospital, Nnewi, and participants gave written informed consent before participating in the study.

### Protocol

Participants were assessed for eligibility to undergo physical tasks using the Physical Activity Readiness Questionnaire. Age as at last birthday, sex as reported by the participants, and routine exercise practice assessed as a “yes” or “no” answer to partaking in structured physical activity of not < 150 min a week for the past 6 months were ascertained. Following the protocol by Chukwuemeka et al.^19^, their weights and heights were taken in kilogrammes using the HANA mechanical bathroom weighing scale (SON 2018, MODEL: FA21418) and metres using a stadiometer/height metre (MODEL: HMS PL), respectively, and an automated blood pressure monitor (Omron-HEM-712) was used to measure the resting and exercise blood pressure and heart rate in mmHg and beats/minute, respectively. The Android mobile version 1.2.4 mobile metronome (Tuner and Metronome) was used and is reliable for exercise protocol^20^.

The exercise was done in the morning, between 8:00 am and 10:00 am and participants wore sports shorts and vests. Before each trial, the participants rested for five minutes in a supine position. After the five-minute rest, the baseline blood pressure and heart rate were recorded in a supine position, and the participants were instructed to stand up. Immediately, blood pressure and heart rate were taken and recorded in standing. The baseline blood pressure and heart rate were measured three times at an interval of 30 s each, and their average score was used for data analysis.

#### Exercise protocol (squatting stress)

All the participants were familiarised with the squatting test before the trials. Participants were asked to squat for two minutes. The participants started the squatting exercise from standing and stopped in a squatting posture for the first minute at the rate of 60 beats set on the metronome; every second is for standing to squat each, making a total of 30 squats per minute. At the end of the first minute, blood pressure and heart rate were recorded in a squatting position, and the participants were asked to stand up immediately. The blood pressure and heart rate were recorded in a standing position. The time interval is 2 min. Then the participants completed another squatting exercise and stopped in a standing posture for the remaining one minute at the same rate. In the end, blood pressure and heart rate were recorded in the standing position, and the participants were asked to squat immediately. The same parameters were recorded in the squatting position. The participants can decide not to complete the squatting exercise if the intensity of the exercise becomes too much for them, as was seen from the person's reaction, which included sweating, breathing heavily, an increased respiratory rate, a reduction in the frequency of exercise, dizziness, and fatigue. The participants were asked to discontinue the exercise, and their blood pressure and heart rate were recorded at the time at which they stopped the exercise. Though all the participants completed the 2 min squatting exercise, three had to repeat the exercise on another day because of their inability to complete the trial the previous day.

### Statistical analysis

The data collected were analysed using Statistical Package for Social Science (SPSS) Version 22.0 and summarised and presented using the frequency table using counts and descriptive statistics of mean and standard deviation. A repeated-measures ANOVA was used to test the squatting exercise effect, and an independent t-test was used to analyse the difference between sex and routine exercise engagement. Shapiro–Wilk tests were used to test the normality assumption; the results were not significant for any of the intervals of measurement of SBP, DBP, and HR. The level of significance was set at 0.05.

### Ethics approval and consent to participate

We want to confirm that all methods used in this study were carried out under the relevant guidelines and regulations as contained in the Helsinki Declaration as amended in Brazil in October 2013. Before the commencement of the study, ethics approval was sought and obtained from the Faculty of Health Sciences Ethics Committee of Nnamdi Azikiwe University, Nnewi (Approval Number: NAU/FHST/2022/MRH84). Also, written informed consent was obtained from the participants before participation.

## Results

Seventy-five (75) undergraduate students (35 male and 40 female) whose mean age was 23 ± 1.2 were involved in this present study, as seen in Table 1. Table 2 shows the characteristics of the participants according to sex, which showed that there was a significant difference in pre- and post-exercise (first and second minute) systolic blood pressure in lying, standing, and squatting positions (*p* < 0.01). There is often a rise in the first-minute post-exercise measures but later falls in the 2 min measures. For instance, there are 134.49 ± 13.66 and 119.15 ± 10.01 mean values for male and female post-squatting exercise systolic blood pressure in the first minute, respectively. The post-squatting exercise diastolic blood pressure in the first minute for males and females is 92.11 ± 12.44 and 86.53 ± 13.56, respectively. There was a comparative fall in the first minute post-squatting exercise diastolic blood pressure in standing, with 91.60 ± 14.40 and 87.80 ± 12.66 mean values for males and females, respectively. On the contrary, the heart rate showed a rise from pre-squatting exercise heart rate in lying at 103.23 ± 13.144 and 103.60 ± 15.36 for males and females when compared to first-minute post-squatting exercise heart rate in standing with values of 107.46 ± 10.842 and 109.53 ± 11.594 for male and female, respectively.

**Table 1:** Description of participants’ characteristics.

Variables	Frequency (N)	Minimum	Maximum	Mean ± Standard deviation
Age	75	18	27	23 ± 2
Weight	75	50	100	68.36 ± 12.03
Height	75	2	2	1.70 ± 0.08
Body mass index (BMI)	75	18	30	23.4 ± 2.9
Pre-exercise systolic blood pressure in lying	75	94	151	112.15 ± 9.51
Pre-exercise diastolic blood pressure in lying	75	62	95	76.57 ± 7.55
Pre-exercise heart rate in lying	75	62	95	81.17 ± 7.40
Pre-exercise systolic blood pressure in standing	75	86	175	111.12 ± 11.26
Pre-exercise diastolic blood pressure in standing	75	60	108	77.04 ± 7.74
Pre-exercise heart rate in standing	75	66	103	86.31 ± 8.06
Post-exercise in squatting				
Systolic blood pressure first minute	75	95	177	126.31 ± 14.07
Systolic blood pressure second minute	71	100	157	126.10 ± 1309
Diastolic blood pressure first minute	75	61	115	89.13 ± 13.09
Diastolic blood pressure second minute	71	61	129	88.01 ± 13.26
Heart rate first minute	75	57	139	103.43 ± 14.28
Heart rate second minute	71	57	130	104.45 ± 15.00
Post-exercise in standing				
Systolic blood pressure first minute	75	95	202	126.05 ± 15.48
Systolic blood pressure second minute	71	91	157	123.46 ± 12.93
Diastolic blood pressure first minute	75	66	123	89.57 ± 13.59
Diastolic blood pressure second minute	71	55	119	87.85 ± 13.83
Heart rate first minute	75	71	139	108.56 ± 11.22
Heart rate second minute	71	62	139	105.85 ± 13.81

**Table 2:** Description of pre- and post-squatting exercise systolic blood pressure, diastolic blood pressure, heart rate, and sociodermographics by sex of the participants.

Variables	Mean ± standard deviation		T	Degree of freedom	*p* value	Mean Difference	95% confidence interval of the difference	
	Male (N = 35)	Female (N = 40)					Lower	Upper
Pre-exercise systolic blood pressure in lying	116 ± 9	108 ± 7	4.51	64.72	< 0.01	8.94	4.98	12.90
Pre-exercise diastolic blood pressure in lying	78 ± 8	76 ± 7	1.28	69.33	0.20	2.25	− 1.25	5.74
Pre-exercise heart rate in lying	80 ± 7	82 ± 7	− 1.10	71.86	0.28	− 1.88	− 5.28	1.53
Pre-exercise systolic blood pressure in standing	117 ± 12	106 ± 7	4.85	52.89	< 0.01	11.35	6.65	16.04
Pre-exercise diastolic blood pressure in standing	79 ± 9	75 ± 7	1.94	63.09	0.06	3.46	− 0.11	7.04
Pre-exercise heart rate in standing	84 ± 8	88 ± 8	− 1.92	71.77	0.06	− 3.52	− 7.18	0.13
Post-exercise in squatting								
Systolic blood pressure first minute	134 ± 14	119 ± 10	5.48	61.59	< 0.01	15.34	9.74	20.93
Systolic blood pressure second minute	132 ± 14	121 ± 10	3.72	56.81	< 0.01	10.86	5.01	16.71
Diastolic blood pressure first minute	92 ± 12	87 ± 14	1.86	72.82	0.07	5.59	− 0.40	11.57
Diastolic blood pressure second minute	91 ± 11	85 ± 14	1.89	68.76	0.06	5.75	− 0.31	11.81
Heart rate first minute	103 ± 13	104 ± 15	− 0.11	72.97	0.91	− 0.37	− 6.93	6.19
Heart rate second minute	103 ± 14	106 ± 16	− 0.93	68.96	0.36	− 3.28	− 10.32	3.77
Post-exercise in standing								
Systolic blood pressure first minute	133 ± 18	120 ± 10	3.54	51.37	< 0.01	12.17	5.27	19.07
Systolic blood pressure second minute	130 ± 13	118 ± 10	4.57	61.24	< 0.01	12.55	7.06	18.04
Diastolic blood pressure first minute	92 ± 14	88 ± 13	1.20	68.12	0.23	3.80	− 2.51	10.11
Diastolic blood pressure second minute	92 ± 13	84 ± 14	2.31	68.96	0.02	7.31	0.99	13.63
Heart rate first minute	107 ± 11	110 ± 12	− 0.80	72.66	0.43	− 2.07	− 7.23	3.10
Heart rate second minute	105 ± 11	106 ± 16	− 0.40	67.09	0.69	− 1.30	− 7.75	5.16
Age (Years)	23 ± 2	22 ± 2	1.48	72.98	0.15	0.67	− 0.24	1.57
Weight (kg)	76 ± 9.37	61.68 ± 10.01	6.40	72.65	0.01	14.33	9.86	18.79
Height (m)	1.75 ± 0.07	1.66 ± 0.07	5.84	70.17	0.01	0.09	0.06	0.13
Body mass index (kg/m^2^)	24.69 ± 2.42	22.30 ± 2.93	3.87	72.77	0.01	2.39	1.16	3.62

**Figure 1:**
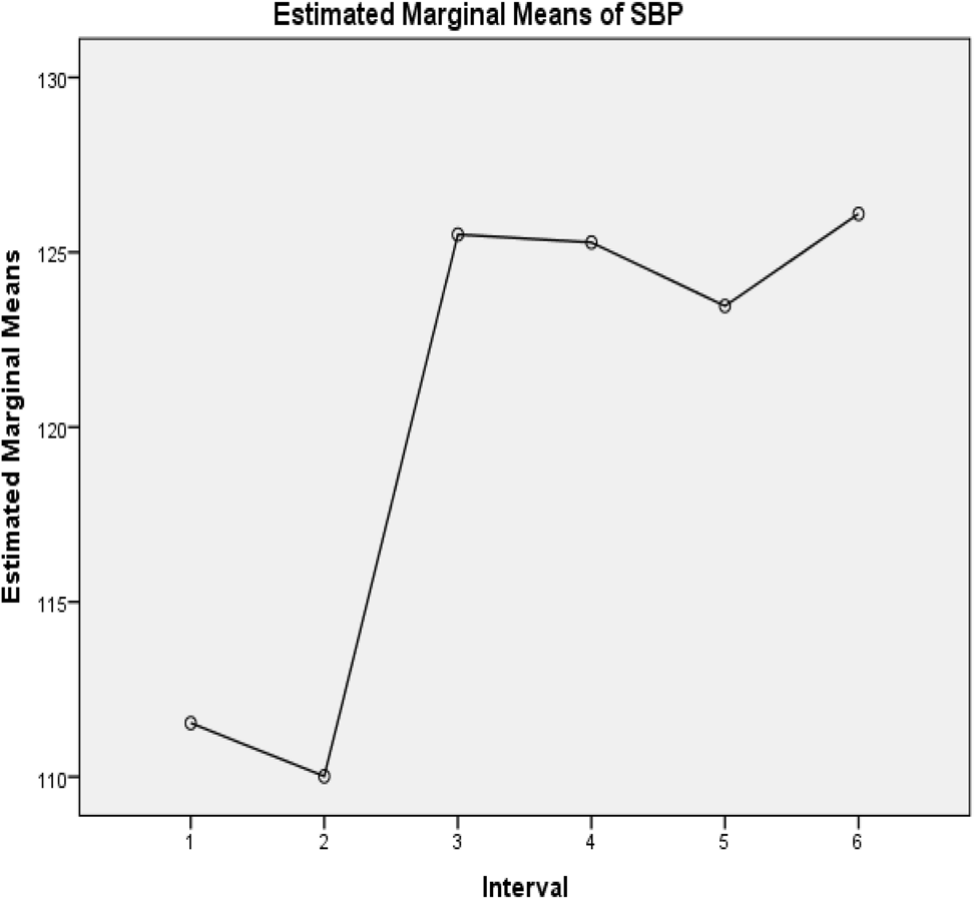
The change in dynamics of the systolic blood pressure from the pre-exercise position in lying to standing and then to the first minute post-exercise in squatting and standing as well as the second minute post-exercise in standing and squatting. *Keys*: SBP = Systolic Blood Pressure in mmHg, 1 = Pre-exercise SBP in lying, 2 = Pre-exercise heart rate in standing, 3 = First minute post-exercise SBP in squatting, 4 = First minute post-exercise SBP in standing, 5 = Second minute post-exercise SBP standing, 6 = Second minute post-exercise SBP in squatting.

**Figure 2:**
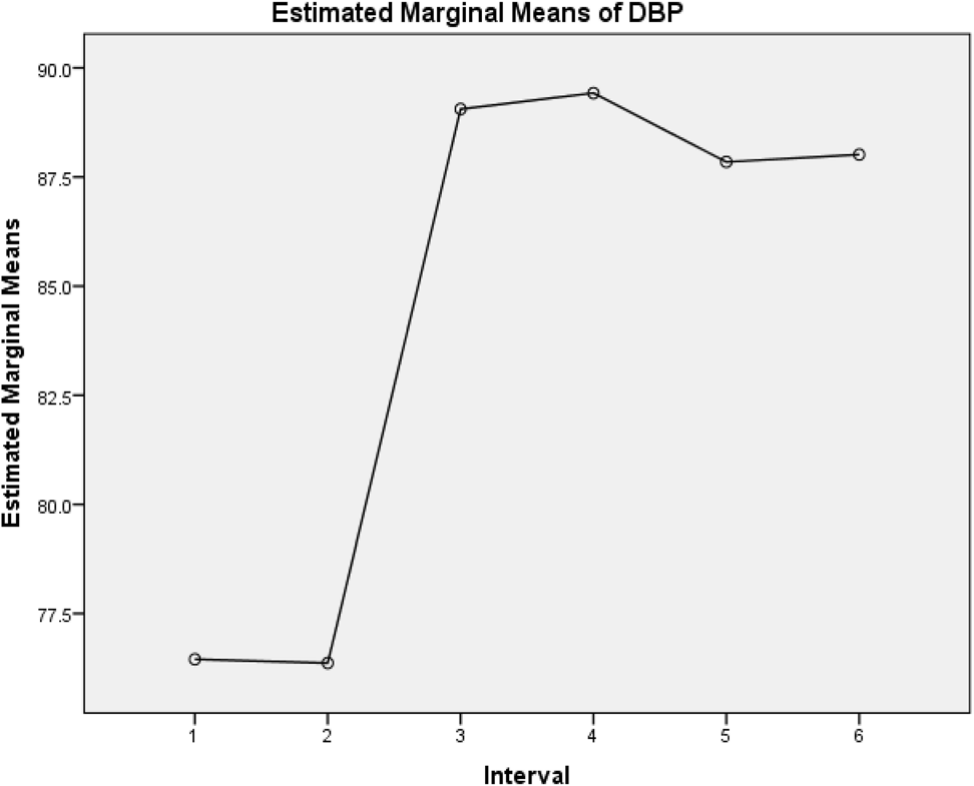
The change in diastolic blood pressure from the pre-exercise position in lying to standing and then to the first minute post-exercise in squatting and standing as well as the second minute post-exercise in standing and squatting. *Keys*: DBP = Diastolic Blood Pressure in mmHg, 1 = Pre-exercise DBP in lying, 2 = Pre-exercise DBP in standing, 3 = First minute post-exercise DBP in squatting, 4 = First minute post-exercise DBP in standing, 5 = Second minute post-exercise DBP standing, 6 = Second minute post-exercise DBP in squatting.

In Table 3, the difference between the pre- and post-exercise systolic blood pressure, diastolic blood pressure, and heart rate in the first and second minutes is presented. The results show a significant difference between pre- and post-squatting exercise blood pressure and heart rate (*p* < 0.01). Before the test, Mauchly’s test was used to examine the sphericity assumption, and the results were significant (χ^2^(2) for SBP = 48.27, DBP = 94.53, and HR = 61.21, *p* ≤ 0.01). Consequently, the degrees of freedom were adjusted with ε = 0.80, 0.66, and 0.77, respectively, using the Greenhouse–Geisser method. Figure 1, 2, and 3 shows the dynamics in change of posture and time interval for systolic blood pressure, diastolic blood pressure, and heart rate respectively.

**Table 3:** Repeated measure ANOVA difference in pre- and post-squatting exercise systolic blood pressure, diastolic blood pressure, and heart rate of the participants.

Variables		Sum of Squares	Degree of freedom	Mean square	F	*p* value
Systolic blood pressure						
Within-subjects	Time	19,751.49	3.96	4989.88	92.40	0.001
	Error	14,963.01	277.08	54		
Between-subjects	Intercept	6,166,842.78	1	6,166,842.78	10,295.30	
	Error	41,929.72	70	599		
Diastolic blood pressure						
Within-subjects	Time	14,162.78	3	4271.31	45.38	0.001
	Error	21,847.22	232.11	94.13		
Between-subjects	Intercept	3,043,605.78	1	3,043,605.78	5905.62	
	Error	36,076.22	70	515.38		
Heart rate						
Within-subjects	Time	48,306.19	3.83	12,621.47	86.74	0.001
	Error	38,985.15	267.91	145.52		
Between-subjects	Intercept	4,134,143.56	1	4,134,143.56	13,466.83	
	Error	21,489.11	70	306.99

**Figure 3:**
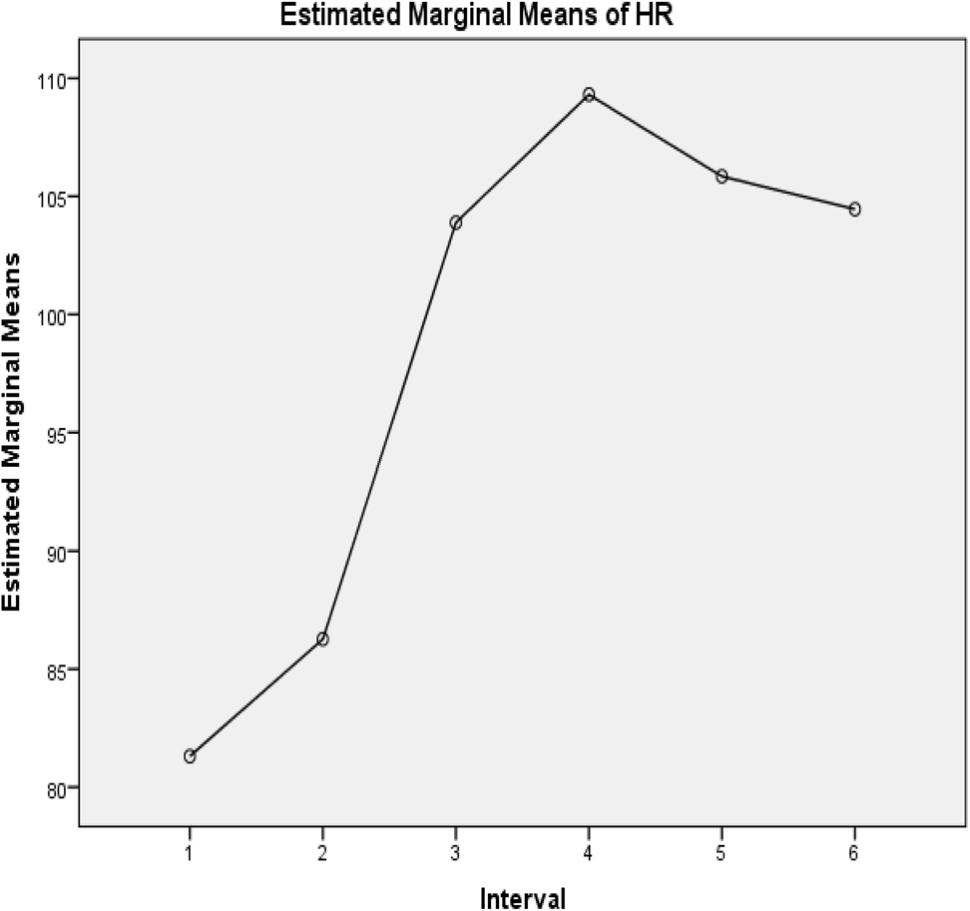
The change in dynamics of the heart rate from the pre-exercise position in lying to standing and then to the first minute post-exercise in squatting and standing as well as the second minute post-exercise in standing and squatting. *Keys*: HR = Heart Rate in beats/minute, 1 = Pre-exercise HR in lying, 2 = Pre-exercise HR in standing, 3 = First minute post-exercise HR in squatting, 4 = First minute post-exercise HR in standing, 5 = Second minute post-exercise HR standing, 6 = Second minute post-exercise HR in squatting.

The results in Table 4 depict the interval where a difference exists pre and post-exercise. There was a significant difference between pre-exercise measures in lying and post post-exercise measures in standing and squatting (*p* < 0.05). Between the first and second minutes post-squatting exercise systolic blood pressure, diastolic blood pressure and heart rate showed no statistically significant difference in the variables (*p* ≥ 0.05).

**Table 4:** Pairwise comparison following Bonferni correction in pre- and post-squatting exercise systolic blood pressure, diastolic blood pressure, and heart rate of the participants.

Variables		Mean difference			Standard error			*P* value			95% confidence interval for difference^b^					
		Systolic	Diastolic	Heart rate	Systolic	Diastolic	Heart rate	Systolic	Diastolic	Heart rate	Systolic		Diastolic		Heart rate	
Posture during measurement											Lower bound	Upper bound	Lower bound	Upper bound	Lower bound	Upper bound
Pre-exercise Lying (1)	2	1.52	0.09	− 4.96*	0.73	0.82	1.14	0.60	1.00	0.01	− 0.69	3.73	− 2.42	2.59	− 8.43	− 1.48
	3	− 13.97*	− 12.61*	− 22.58*	1.14	1.45	1.71	0.01	0.01	0.01	− 17.43	− 10.52	− 17.00	− 8.22	− 27.76	− 17.39
	4	− 13.75*	− 12.97*	− 28.00*	1.07	1.59	1.40	0.01	0.01	0.01	− 17.01	− 10.49	− 17.44	− 8.51	− 32.25	− 23.75
	5	− 11.93*	− 11.39*	− 24.54*	1.25	1.50	1.59	0.01	0.01	0.01	− 15.73	− 8.13	− 15.96	− 6.83	− 29.36	− 19.71
	6	− 14.56*	11.56*	− 23.14*	1.26	1.41	1.60	0.01	0.01	0.01	− 18.38	− 10.74	− 15.86	− 7.27	− 27.99	− 18.29
Pre-exercise Standing (2)	1	− 1.52	− 0.085	4.96*	0.73	0.82	1.14	0.60	1.00	0.01	− 3.73	0.69	− 2.59	2.42	1.484	8.43
	3	− 15.49*	− 12.69*	− 17.62*	0.97	1.43	2.11	0.01	0.01	0.01	− 18.43	− 12.56	− 17.02	− 8.36	− 24.04	− 11.20
	4	− 15.27*	− 13.06*	− 23.04*	1.01	1.43	1.62	0.01	0.01	0.01	− 18.34	− 12.19	− 17.39	− 8.73	− 27.97	− 18.12
	5	− 13.45*	− 11.48*	− 19.58*	1.06	1.51	1.77	0.01	0.01	0.01	− 16.68	− 10.23	− 16.08	− 6.88	− 24.97	− 14.19
	6	− 16.09*	− 11.65*	− 18.18*	1.19	1.45	2.04	0.01	0.01	0.01	− 19.69	− 12.48	− 16.07	− 7.23	− 24.37	− 12.00
Post-exercise Squatting first minute (3)	1	13.97*	12.61*	22.58*	1.14	1.45	1.71	0.01	0.01	0.01	10.52	17.43	8.22	17.00	17.39	27.76
	2	15.49*	12.69*	17.62*	0.97	1.43	2.11	0.01	0.01	0.01	12.56	18.43	8.36	17.02	11.20	24.04
	4	0.23	− 0.37	− 5.42*	0.98	0.96	1.55	1.00	1.00	0.01	− 2.76	3.21	− 3.28	2.55	− 10.14	− 0.70
	5	2.04	1.21	− 1.95	0.098	1.46	2.20	0.61	1.00	1.00	− 0.93	5.01	− 3.21	5.64	− 8.64	4.72
	6	− 0.59	1.04	− 0.56	1.09	1.18	1.73	1.00	1.00	1.00	− 3.90	2.72	− 2.55	4.64	− 5.81	4.69
Post-exercise Standing first minute (4)	1	13.75*	12.97*	28.00*	1.07	1.47	1.40	0.01	0.01	0.01	10.49	17.01	8.51	17.44	23.75	32.25
	2	15.27*	13.06*	23.042*	1.01	1.43	1.62	0.01	0.01	.000	12.19	18.33	8.73	17.39	18.12	27.97
	3	− 0.23	0.37	5.42*	0.98	0.96	1.55	1.00	1.00	.013	− 3.21	2.76	− 2.55	3.28	0.70	10.14
	5	1.82	1.58	3.47	1.23	1.34	1.96	1.00	1.00	1.00	− 1.91	5.54	− 2.49	5.64	− 2.50	9.424
	6	− 0.82	1.41	4.86	1.32	1.30	1.85	1.00	1.00	0.16	− 4.83	3.20	− 2.53	5.35	− 0.77	10.49
Post-exercise Standing second minute (5)	1	11.93*	11.39*	24.54*	1.25	1.50	1.59	0.01	0.01	0.01	8.13	15.73	6.83	15.96	19.71	29.36
	2	13.45*	11.48*	19.58*	1.06	1.51	1.77	0.01	0.01	0.01	10.23	16.68	6.88	16.08	14.19	24.97
	3	− 2.04	− 1.21	1.96	0.98	1.46	2.20	0.60	1.00	1.00	− 5.01	0.93	− 5.64	3.21	− 4.72	8.64
	4	− 1.82	− 1.58	− 3.47	1.23	1.34	1.96	1.00	1.00	1.00	− 5.54	1.91	− 5.64	2.49	− 9.42	2.50
	6	− 2.63	− 0.17	1.39	1.05	0.91	1.99	0.22	1.00	1.00	− 5.84	.57	− 2.94	2.60	− 4.64	7.43
Post-exercise Squatting second minute (6)	1	14.56*	11.56*	23.14*	1.26	1.41	1.60	0.01	0.01	0.01	10.74	18.38	7.27	15.86	18.29	27.99
	2	16.09*	11.65*	18.18*	1.19	1.45	2.04	0.01	0.01	0.01	12.48	19.69	7.23	16.07	12.00	24.37
	3	0.59	− 1.04	0.56	1.09	1.18	1.73	1.00	1.00	1.00	− 2.72	3.90	− 4.64	2.55	− 4.69	5.81
	4	0.82	− 1.41	− 4.86	1.32	1.30	1.85	1.00	1.00	0.16	− 3.20	4.83	− 5.35	2.53	− 10.49	0.77
	5	2.63	0.17	− 1.39	1.05	0.91	1.99	0.22	1.00	1.00	− 0.57	5.84	− 2.60	2.94	− 7.43	4.64

Lastly, Table 5 shows the characteristics of the participants according to routine exercise engagement, which showed that there was a significance difference in pre- and post-exercise (first and second minute) systolic blood pressure in lying, standing, and squatting positions (*p* < 0.05), except for post-exercise systolic blood pressure in squatting at the first minute (*p* = 0.05).

**Table 5:** Difference in pre- and post-squatting exercise systolic blood pressure, diastolic blood pressure, and heart rate by routine exercise engagement of the participants.

Variables	Mean ± standard deviation		T	Degree of freedom	*p* value	Mean difference	95% confidence interval of the difference	
	Routine exercises (N = 36)	No routine exercises (N = 39)					Lower	Upper
Pre-exercise systolic blood pressure in lying	116 ± 8	109 ± 10	3.43	70.61	0.01	6.98	2.93	11.04
Pre-exercise diastolic blood pressure in lying	77 ± 8	76 ± 7	0.56	69.87	0.58	0.98	− 2.53	4.450
Pre-exercise heart rate in lying	81 ± 7	81 ± 8	− 0.04	73.00	0.97	− 0.07	− 3.49	3.35
Pre-exercise systolic blood pressure in standing	114 ± 7	108 ± 13	2.58	59.80	0.01	6.34	1.42	11.26
Pre-exercise diastolic blood pressure in standing	77 ± 7	77 ± 8	− 0.37	72.55	0.71	− 0.67	− 4.23	2.90
Pre-exercise heart rate in standing	86 ± 7	86 ± 9	− 0.20	72.39	0.84	− 0.38	− 4.09	3.34
Post-exercise in squatting								
Systolic blood pressure first minute	131 ± 14	122 ± 13	2.70	72.50	0.01	8.44	2.22	14.66
Systolic blood pressure second minute	131 ± 13	121 ± 11	3.49	67.54	0.01	10.06	4.31	15.80
Diastolic blood pressure first minute	88 ± 13	90 ± 14	− 0.47	73.00	0.64	− 1.43	− 7.55	4.69
Diastolic blood pressure second minute	89 ± 13	87 ± 13	0.84	68.98	0.41	2.62	− 3.63	8.87
Heart rate first minute	101 ± 14	106 ± 15	− 1.33	72.94	0.19	− 4.35	− 10.88	2.18
Heart rate second minute	103 ± 15	106 ± 15	− 0.78	68.50	0.44	− 2.77	− 9.90	4.36
Post-exercise in standing								
Systolic blood pressure first minute	130 ± 13	123 ± 17	2.04	72.04	0.05	7.11	0.18	14.04
Systolic blood pressure second minute	129 ± 14	118 ± 9	3.94	62.30	0.01	10.95	5.39	16.51
Diastolic blood pressure first minute	90 ± 14	90 ± 13	− 0.23	71.96	0.82	− 0.73	− 7.04	5.58
Diastolic blood pressure second minute	89 ± 14	86 ± 13	0.89	68.91	0.38	2.91	− 3.65	9.46
Heart rate first minute	108 ± 11	109 ± 12	− 0.33	72.92	0.74	− 0.86	− 6.05	4.33
Heart rate second minute	107 ± 14	105 ± 14	0.44	68.55	0.66	1.44	− 5.14	8.02
Age (Years)	23 ± 2	22 ± 2	1.64	72.96	0.11	0.74	− 0.16	1.65
Weight (kg)	73.67 ± 11.48	63.46 ± 10.45	4.01	70.84	0.01	10.21	5.14	15.28
Height (m)	1.73 ± 0.09	1.68 ± 0.07	2.73	66.35	0.01	0.05	0.01	0.09
Body mass index (kg/m^2^)	24.51 ± 2.64	22.40 ± 2.87	3.30	73.00	0.01	2.12	0.85	3.38

## Discussions

This study was aimed at determining the effects of squatting on selected cardiovascular parameters among undergraduates. The main finding from the study results revealed a statistically significant difference between pre- and post-exercise blood pressure and heart rate in the first and second minutes. This implies that the squatting test has a marked effect on cardiovascular parameters and confirms the use of squatting for cardiovascular autonomic function tests. Kate et al.^1^ reported similarly that there is a significant change in systolic and diastolic blood pressure and heart rate after squatting trials among undergraduate medical students in India. This is explained to be because the shift from squatting to standing imposes major orthostatic stress, leading to rapid and large changes in arterial blood pressure and heart rate, which are utilised to assess the baroreceptor reflex and integrity of the autonomic nervous system in man^21^. In a similar study carried out by Philips and Scheen^17^ among patients with hypo-adrenergic orthostatic hypotension, they showed that there occurs a profound hemodynamic change, which is a rise in systemic arterial blood pressure and a rise in arterial oxygen saturation, most occurring during the first 30 s because squatting impedes the venous return from the legs. Tschakovsky et al.^18^ also explained that the reason behind the rise in blood pressure, which causes a significant change, is that squatting increases venous return as a result of muscular pumping in the legs, which enhances cardiac output (CO). The kinking of femoral arteries, which may increase peripheral resistance, does not appear to play a significant role, and thus the increase in blood pressure is explained by increased preload caused by an augmentation of venous return. Hanson et al.^8^ also supported the explanation that these significant increases in blood pressure and heart rate during squatting are caused by increased venous return and are not dependent on cardiac innervation. Notwithstanding, Wieling et al.^22^ pointed out in a more convincing finding that two factors are involved in the rapid fall in pressure, including a sudden decrease in total peripheral resistance in the legs due to the ischemic effect of squatting that enables a very rapid inflow of arterial blood in the legs and due to a marked pooling of blood in the venous vessels in the legs and abdomen that have been squeezed in the squatting position, resulting in a decrease in venous return and thereby in cardiac output.

Meanwhile, findings from the study by Tschakovsky et al.^18^ reported on the contrary that heart rate decreased significantly after squatting trials. The difference in their report might be attributed to their use of a low sample size and more females than males, as their study was among young, healthy subjects (2 males and 15 females) with an average age of 22.5 ± 1.0 years. When comparing squatting to standing with lying to standing, only the pre-exercise heart rate was statistically significantly different, confirming the squatting test as a more dynamic postural manoeuvre to study baroreflex sensitivity (19). Gambassi et al.^23^ assert that exercise incorporating squatting can be considered functional for activities of daily life, thus prioritizing it in their protocol.

A study by Stewart and Clarke^24^ and Wijnen et al.^25^ reported a similar increase only in heart rate between the lying and squatting tests. This difference in the two tests is clearly explained to be due to the different physiological autonomic mechanisms that they trigger. The mechanisms underlying the fall in blood pressure from lying to standing included the muscle pump, rapid locally mediated vasodilatation effects (both factors in the active muscles involved in the effort of standing), and cardiopulmonary receptor-mediated systemic sympathetic withdrawal in response to sudden increases in right atrial pressure^22^. This research alerts the clinician to common, though neglected, conditions that occur with an active change of posture during treatment sessions. The results are expected to guide physiotherapists and other health care workers on the level of exposure of their patients to certain exercises and also to put into consideration the level of changes in blood pressure and heart rate that will take place when putting the patients from lying to standing to squatting and back to standing. It also adds a clear explanation to the underlying mechanism in understanding the pathophysiology and provides evidence for advising patients to rise slowly, especially at night, from supine against orthostatic hypotension.

The statistically significant influence of exercise engagement on the systolic blood pressure in pre- and post-exercise measures calls for in-depth cause-and-effect research on the reason for this. We could posit that this change may be due to increased exercise tolerance due to the target cardiovascular endurance, as this is supported by research findings^26^.

Although this study's finding adds an important update from the Nigerian and African contexts where data has been scarce, certain limitations apply to its findings that could affect the generalisation of its findings. First, this is a Unicenter study among the Igbo ethnic group only, thus it might not account for possible environmental differences. It also recruited only healthy students, but many cases of orthotic hypotension and postural syncope are often seen in unhealthy patients. Thus, future research is recommended in other settings and for special categories of patients, especially those with neurological diagnoses.

In conclusion, squatting trials among undergraduates revealed some statistically significant changes, especially between the blood pressure and heart parameters obtained in the standing position compared to lying and those measured after the first minute and second minute of squatting and standing. Further studies on the effects of squatting on selected cardiovascular parameters should be carried out among special clinical categories, like hypertensive patients, as more heightened manifestations may be found.

## Data Availability

The datasets used and/or analysed during the current study are available from the corresponding author upon reasonable request.
